# Supplementary material for: Pharmacological Screening of Kv7.1 and Kv7.1/KCNE1 Activators as Potential Antiarrhythmic Drugs in the Zebrafish Heart
Source: Int J Mol Sci. 2023 Jul 28;24(15):12092. doi: 10.3390/ijms241512092 (PMC10418701; doi:10.3390/ijms241512092)
Supplement: Supplementary file 1 [file ijms-24-12092-s001.zip › ijms-2519475-supplementary.pdf]

Supplementary Materials for

Pharmacological Screening of Kv7.1 and Kv7.1/KCNE1 Activators as Potential Antiarrhythmic Drugs in the Zebrafish Heart

Alicia De la Cruz [1], Xiaoan Wu [1], Quinn C. Rainer [1], Irene Hiniesto-Iñigo [2], Marta E. Perez [1], Isak Edler [1,2], Sara I. Liin [2] and H. Peter Larsson [1,*]

[1] Department of Physiology and Biophysics, Miller School of Medicine, University of Miami, 1600 NW 10th Avenue, Miami, FL 33136, USA

[2] Department of Biomedical and Clinical Sciences, Linköping University, SE-581 85 Linköping, Sweden

* Correspondence: plarsson@med.miami.edu; Tel.: +1-305-243-1021

This PDF file includes:

Figures S1 to S6

Tables S1 to S2

Supplementary Figures

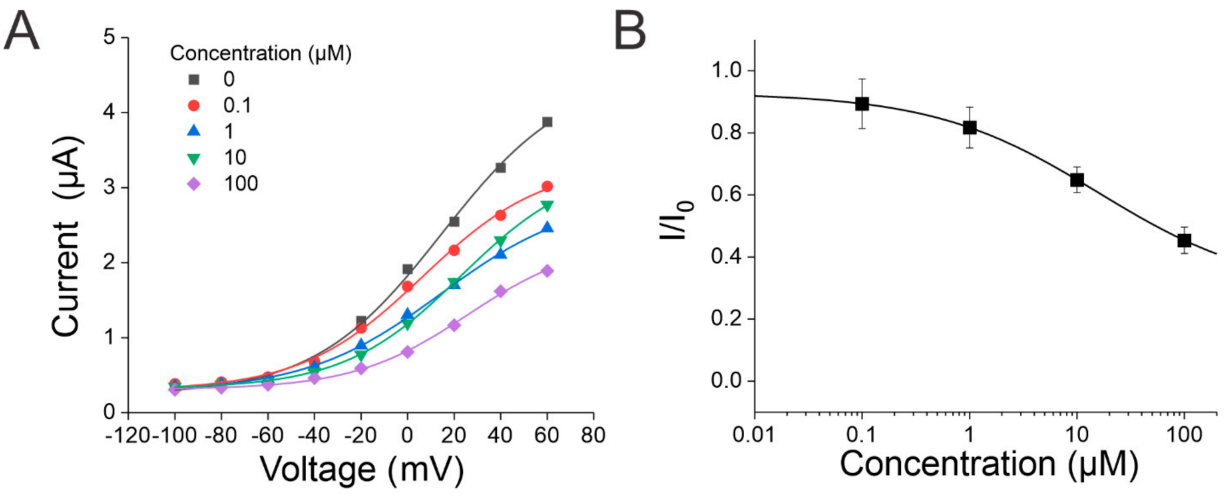

Figure S1. JNJ303 inhibits zebrafish I_{Ks} currents recorded in *Xenopus laevis* oocytes only at very high concentrations. (A) Representative current measurements of zebrafish Kv7.1/KCNE1 before and after the application of JNJ303 with different concentrations (from 0.01 to 100 μM). From a holding potential of −80 mV followed by a hyperpolarizing prepulse to −140 mV, currents were elicited to potential of +40 mV followed by a tail voltage of −40 mV. (**B)** Concentration-response relationship for the inhibition I_{Ks} by JNJ303. Inhibition was determined as a percent change in current amplitude at 0 mV. The data were fitted with the Hill equation (IC50 > 100 μM, n=3).

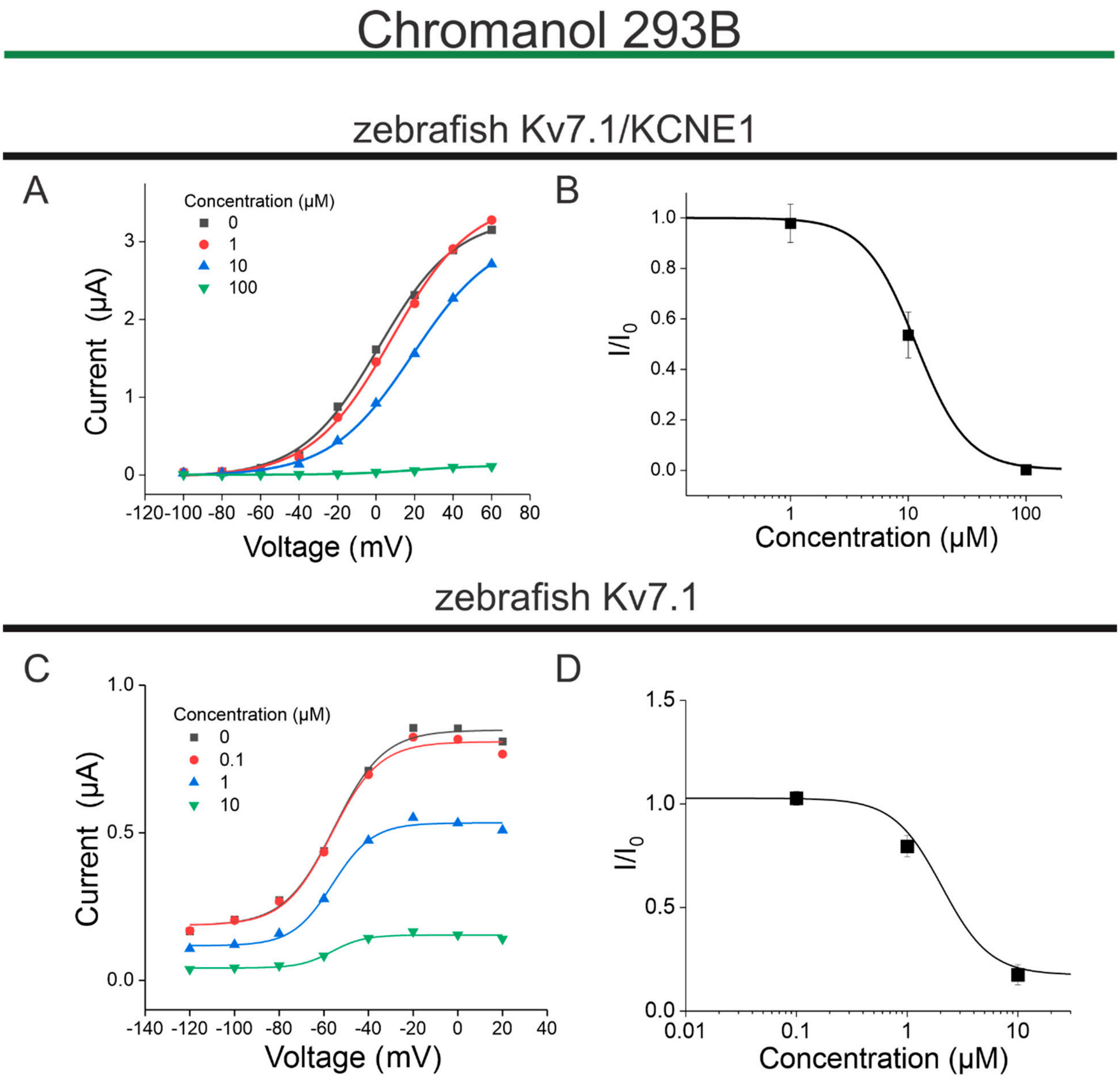

Figure S2. Chromanol 293B inhibits zebrafish Kv7.1/KCNE1 and Kv7.1 currents recorded in *Xenopus laevis* oocytes. (A,C) Representative current measurements of zebrafish Kv7.1/KCNE1 and Kv7.1, respectively, before and after the application of chromanol 293B with different concentrations (from 1 to 100 μM). From a holding potential of −80 mV followed by a hyperpolarizing prepulse to −140 mV, currents were elicited to potential of 0 mV followed by a tail voltage of −40 mV. **(B,D)** Concentration-response relationship for the inhibition Kv7.1/KCNE1 and Kv7.1 by chromanol 293B. Inhibition was determined as a percent change in current amplitude at 0 mV. The data were fitted with the Hill equation (IC50 = 11.6 ± 0.01 μM, n=5, and IC50 = 2.1 ± 0.01 μM, n=5, for Kv7.1/KCNE1 and Kv7.1, respectively).

DHA-Gly

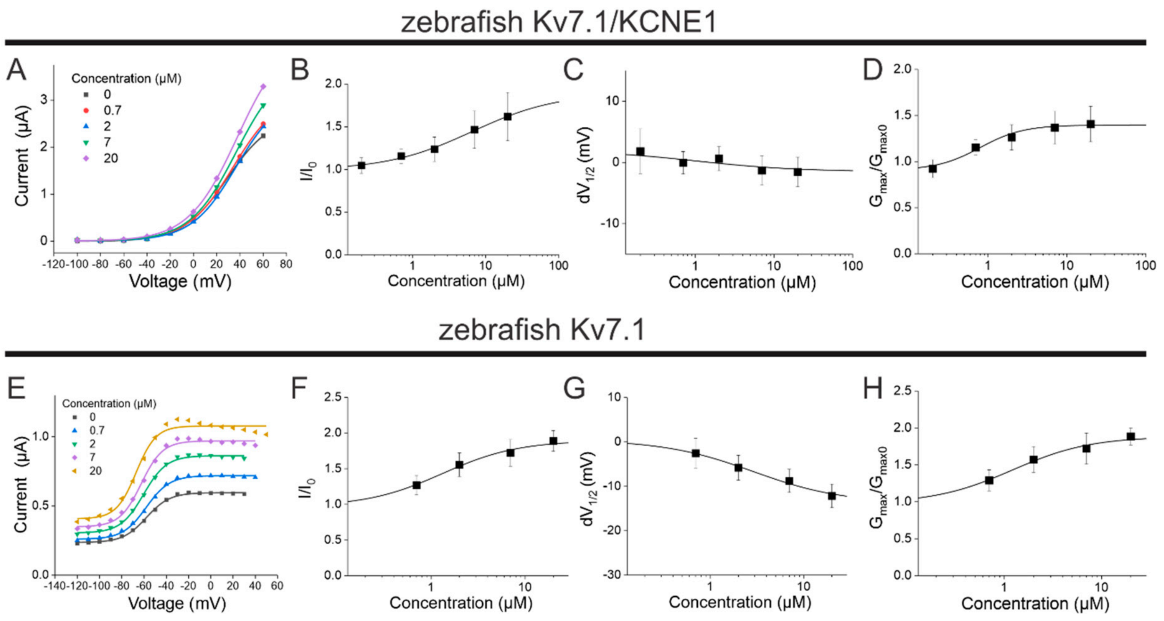

Figure S3. DHA-Gly does not significantly increase zebrafish I_{Ks} currents recorded in *Xenopus laevis* oocytes. (A) Representative current measurements of zebrafish Kv7.1/KCNE1 before and after the application of DHA-Gly with different concentrations (from 0.7 to 20 µM). From a holding potential of −80 mV followed by a hyperpolarizing prepulse to −140 mV, currents were elicited to a potential of +40 mV followed by a tail voltage of −40 mV. **(B–D)** Concentration-response relationship in the **(B)** current amplitude, **(C)** voltage dependence of activation ($\Delta V_{1/2}$) and **(D)** changes in maximal conductance (Gmax) for Kv7.1/KCNE1 induced by DHA-Gly (n = 11, mean ± SEM, Table S1). **E)** Representative current measurements of zebrafish Kv7.1 before and after the application of DHA-Gly with different concentrations (from 0.7 to 20 µM) **(F–H)** Concentration-response relationship in the **(F)** current amplitude, **(G)** voltage dependence of activation ($\Delta V_{1/2}$) and **(H)** changes in maximal conductance (Gmax) for Kv7.1 induced by DHA-Gly (n = 5, mean ± SEM, Table S1). The data were fitted with the Hill equation (IC50 = 6.5 ± 5.6 µM, n=11, and IC50 = 1.3 ± 3.4 µM, n=4, for Kv7.1/KCNE1 and Kv7.1, respectively)

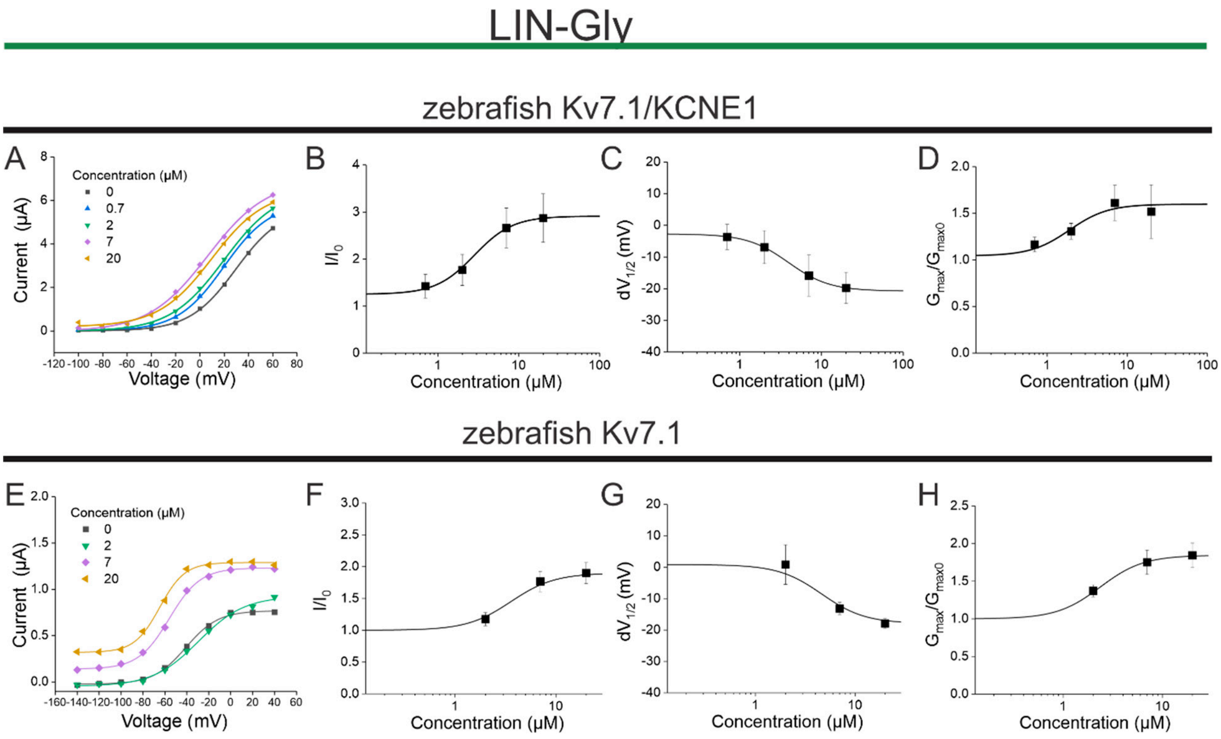

Figure S4: LIN-Gly increases significantly zebrafish Kv7.1/KCNE1 and Kv7.1 currents recorded in *Xenopus laevis* oocytes. (A) Representative current measurements of zebrafish Kv7.1/KCNE1 before and after the application of LIN-Gly with different concentrations (from 0.7 to 20 µM). From a holding potential of −80 mV followed by a hyperpolarizing prepulse to −140 mV, currents were elicited to a potential of +40 mV followed by a tail voltage of −40 mV. **(B–D)** Concentration-response relationship in the **(B)** current amplitude, **(C)** voltage dependence of activation ($\Delta V_{1/2}$) and **(D)** changes in maximal conductance (Gmax) for Kv7.1/KCNE1 induced by LIN-Gly (n = 7, mean ± SEM, Table S1). **E)** Representative current measurements of zebrafish Kv7.1 before and after the application of LIN-Gly with different concentrations (from 0.7 to 20 µM) **(F–H)** Concentration-response relationship in the **(F)** current amplitude, **(G)** voltage dependence of activation ($\Delta V_{1/2}$) and **(H)** changes in maximal conductance (Gmax) for Kv7.1 induced by LIN-Gly (n = 6, mean ± SEM, Table S2). The data were fitted with the Hill equation (IC50 = 2.8 ± 1.7 µM, n=7, and IC50 = 3.6 ± 0.01 µM, n=6, for Kv7.1/KCNE1 and Kv7.1, respectively)

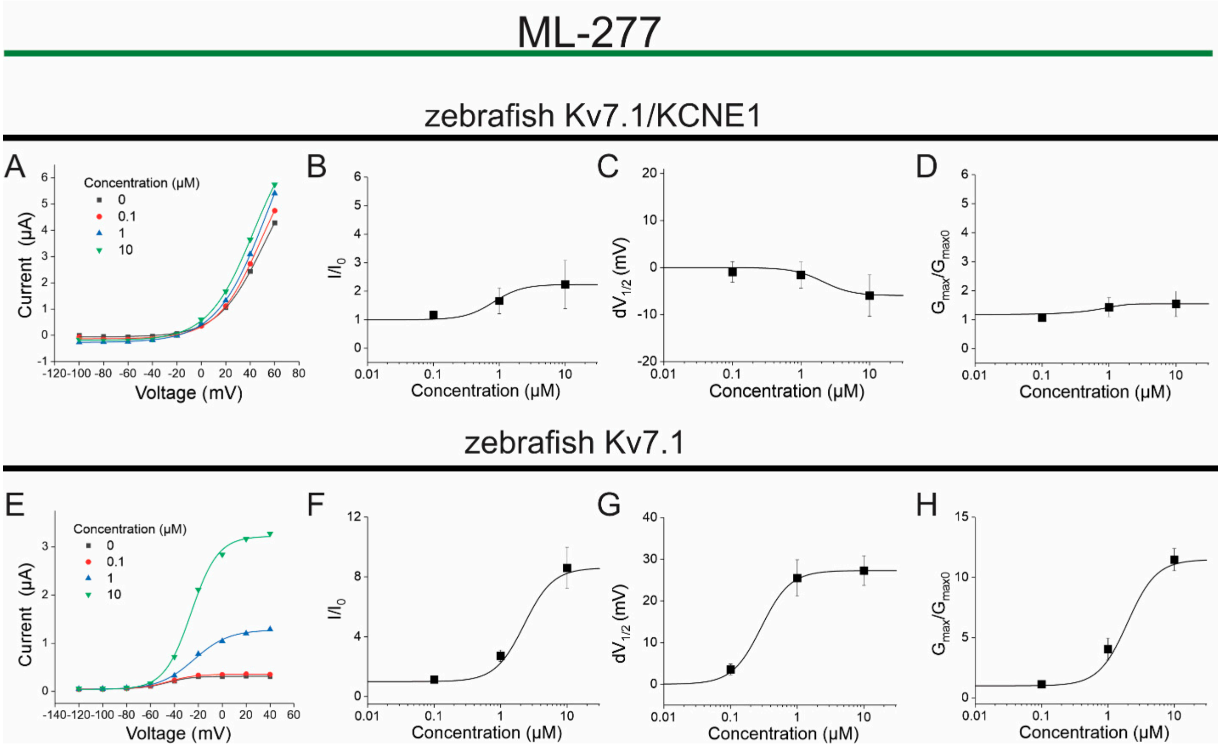

Figure S5. ML-277 effects on zebrafish Kv7.1/KCNE1 and Kv7.1 currents recorded in *Xenopus laevis* oocytes. (A) Representative current measurements of zebrafish Kv7.1/KCNE1 before and after the application of ML-277 with different concentrations (from 0.1 to 10 μM). From a holding a potential of −80 mV followed by a hyperpolarizing prepulse to −140 mV, currents were elicited to potential of +40 mV followed by a tail voltage of −40 mV. **(B–D)** Concentration-response relationship in the **(B)** current amplitude, **(C)** voltage dependence of activation ($\Delta V_{1/2}$) and **(D)** changes in maximal conductance (Gmax) for Kv7.1/KCNE1 induced by ML-277 (n = 6, mean ± SEM, Table S1). **E)** Representative current measurements of zebrafish Kv7.1 before and after the application of ML-277 with different concentrations (from 0.1 to 10 μM) **(F–H)** Concentration-response relationship in the **(F)** current amplitude, **(G)** voltage dependence of activation ($\Delta V_{1/2}$) and **(H)** changes in maximal conductance (Gmax) for Kv7.1 induced by ML-277 (n = 6, mean ± SEM, Table S2). The data were fitted with the Hill equation (IC50 = 0.8 ± 0.01 μM, n=6, and IC50 = 2.3 ± 0.01 μM, n=6, for Kv7.1/KCNE1 and Kv7.1, respectively)

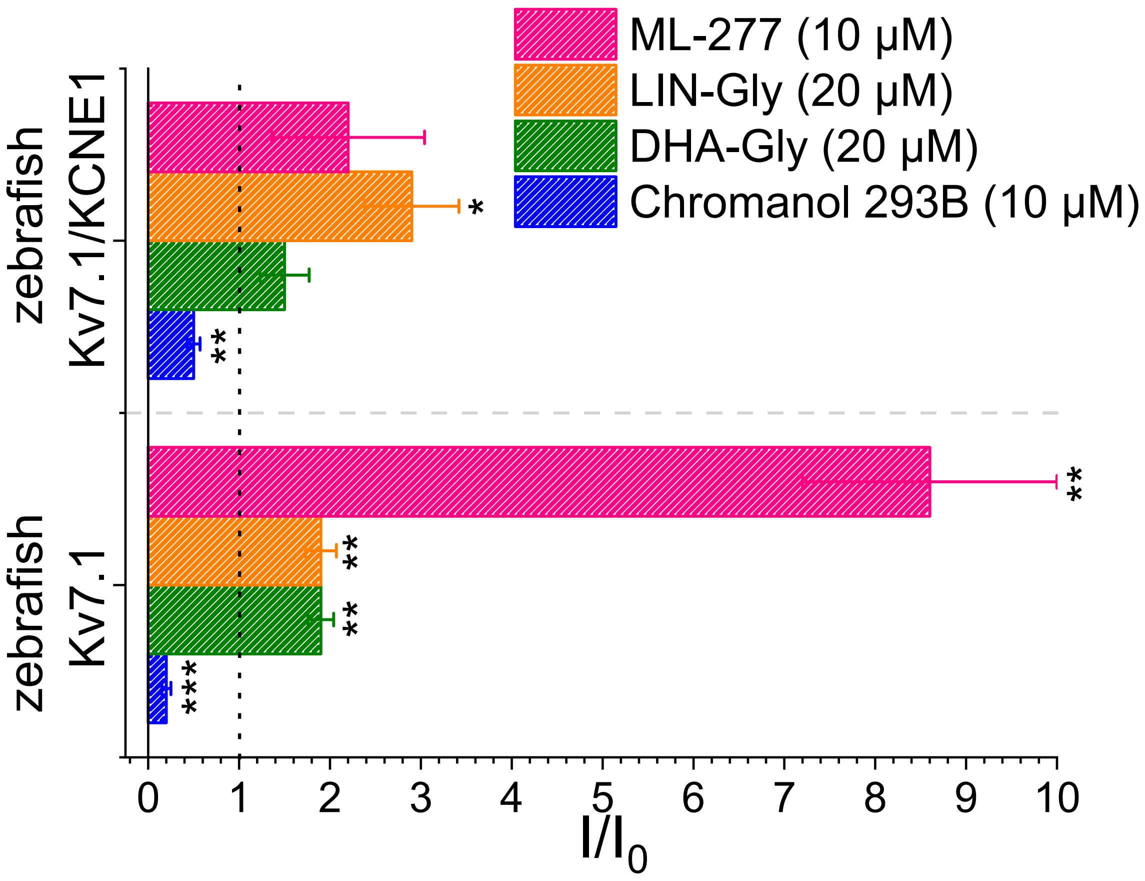

Figure S6: Summary of zebrafish Kv7.1/KCNE1 and Kv7.1 blockers and activators effects recorded in *Xenopus laevis* oocytes. The summary was performed from data of the zebrafish Kv7.1/KCNE1 and Kv7.1 blocker (chromanol 293B) and activators (DHA-Gly, LIN-Gly, and ML-277). Bars are showing the effects of these compounds on the I/I_0 for zebrafish Kv7.1/KCNE1 and Kv7.1 recorded in *Xenopus laevis* oocytes (mean ± SEM, *p < 0.05, **p< 0.01, and ***p< 0.001).

Supplementary Tables

Supplementary Table S1. Zebrafish I_{Ks} measurements in Xenopus laevis oocytes for I_{Ks} inhibitors, and I_{Ks} activator and PUFA analogues.

	$\Delta V_{0.5}$	*(n, p)*	*Gmax/Gmax0*	*(n, p)*	*I/I0*	*(n, p)*
C293B (10 µM)	10.9 ± 2.9	n = 5, $p < 0.05$	0.8 ± 0.07	n = 5, NS, $p > 0.05$	0.5 ± 0.07	n = 5, $p < 0.01$
ML-277 (1 µM)	−1.6 ± 2.8	n = 6, NS, $p > 0.05$	1.4 ± 0.32	n = 6, NS, $p > 0.05$	1.7 ± 0.45	n = 6, NS, $p > 0.05$
DHA-Gly (20 µM)	−0.5 ± 2.4	n = 11, NS, $p > 0.05$	1.4 ± 0.20	n = 11, NS, $p > 0.05$	1.5 ± 0.27	n = 11, NS, $p > 0.05$
LIN-Gly (20 µM)	−19.8 ± 4.9	n = 7, $p < 0.01$	1.5 ± 0.29	n = 7, NS, $p > 0.05$	2.9 ± 0.52	n = 7, $p < 0.05$

Supplementary Table S2. Zebrafish Kv7.1 measurements in Xenopus laevis oocytes for Kv7.1 inhibitors, and Kv7.1 activator and PUFA analogues.

	$\Delta V_{0.5}$	*(n, p)*	*Gmax/Gmax0*	*(n, p)*	*I/I0*	*(n, p)*
C293B (10 µM)	2.0 ± 1.1	n = 5, NS, $p > 0.05$	0.2 ± 0.05	n = 5, $p < 0.001$	0.2 ± 0.05	n = 5, $p < 0.001$
ML-277 (1 µM)	26.5 ± 4.6	n = 6, $p < 0.01$	4.0 ± 0.76	n = 6, $p < 0.05$	2.7 ± 0.38	n = 6, $p < 0.01$
DHA-Gly (20 µM)	−12.2 ± 2.7	n = 4, $p < 0.05$	1.9 ± 0.11	n = 4, $p < 0.01$	1.9 ± 0.14	n = 4, $p < 0.01$
LIN-Gly (20 µM)	−17.9 ± 1.5	n = 6, $p < 0.001$	1.8 ± 0.16	n = 6, $p < 0.01$	1.9 ± 0.17	n = 6, $p < 0.01$
